# Implementation of linked data in the life sciences at BioHackathon 2011

**DOI:** 10.1186/2041-1480-6-3

**Published:** 2015-01-07

**Authors:** Kiyoko F Aoki-Kinoshita, Akira R Kinjo, Mizuki Morita, Yoshinobu Igarashi, Yi-an Chen, Yasumasa Shigemoto, Takatomo Fujisawa, Yukie Akune, Takeo Katoda, Anna Kokubu, Takaaki Mori, Mitsuteru Nakao, Shuichi Kawashima, Shinobu Okamoto, Toshiaki Katayama, Soichi Ogishima

**Affiliations:** Department of Bioinformatics, Faculty of Engineering, Soka University, 1-236 Tangi-machi, Hachioji, Tokyo, 192-8577 Japan; Laboratory of Protein Informatics, Laboratory of Protein Databases, and Protein Data Bank Japan, Research Center for Structural and Functional Proteomics, Institute for Protein Research, Osaka University, 3-2 Yamadaoka, Suita, Osaka, 565-0871 Japan; Center for Knowledge Structuring, The University of Tokyo, 7-3-1 Hongo, Bunkyo-ku, Tokyo, Japan; National Institute of Biomedical Innovation, 7-6-8 Asagi Saito, Ibaraki-City, Osaka, 567-0085 Japan; DNA Data Bank of Japan, National Institute of Genetics, Yata 1111, Mishima, Shizuoka, 411-8540 Japan; Next Generation Systems Core Function Unit, Eisai Product Creation Systems, Eisai Co., Ltd, Tsukuba, Ibaraki, Japan; Database Center for Life Science, Research Organization of Information and Systems, 178-4-4 Wakashiba, Kashiwa-shi, Chiba, 277-0871 Japan; Department of Bioclinical informatics, Tohoku Medical Megabank Organization, Tohoku University, Seiryo-cho 4-1, Aoba-ku, Sendai-shi Miyagi, 980-8575 Japan

**Keywords:** Semantic Web, Data integration, PDBj, DDBJ, Glycobiology, Alzheimer’s disease, Faceted search interface

## Abstract

**Background:**

Linked Data has gained some attention recently in the life sciences as an effective way to provide and share data. As a part of the Semantic Web, data are linked so that a person or machine can explore the web of data. Resource Description Framework (RDF) is the standard means of implementing Linked Data. In the process of generating RDF data, not only are data simply linked to one another, the links themselves are characterized by ontologies, thereby allowing the types of links to be distinguished. Although there is a high labor cost to define an ontology for data providers, the merit lies in the higher level of interoperability with data analysis and visualization software. This increase in interoperability facilitates the multi-faceted retrieval of data, and the appropriate data can be quickly extracted and visualized. Such retrieval is usually performed using the SPARQL (SPARQL Protocol and RDF Query Language) query language, which is used to query RDF data stores. For the database provider, such interoperability will surely lead to an increase in the number of users.

**Results:**

This manuscript describes the experiences and discussions shared among participants of the week-long BioHackathon 2011 who went through the development of RDF representations of their own data and developed specific RDF and SPARQL use cases. Advice regarding considerations to take when developing RDF representations of their data are provided for bioinformaticians considering making data available and interoperable.

**Conclusions:**

Participants of the BioHackathon 2011 were able to produce RDF representations of their data and gain a better understanding of the requirements for producing such data in a period of just five days. We summarize the work accomplished with the hope that it will be useful for researchers involved in developing laboratory databases or data analysis, and those who are considering such technologies as RDF and Linked Data.

## Introduction

As technologies in the life sciences advance among various -omics fields in the post-genomic age, increasing amounts of a wide variety of data are being generated, making it difficult to query and find relationships between the data. Currently, many of the databases that allow data to be downloaded often provide them in their own proprietary format or as tab- or comma-delimited text files. Incorporating such data requires much data manipulation and integration, which is usually difficult for most researchers. In order to process such data, most biologists would use Excel and would probably need to write scripts to find matching data across different data files, if not done manually. Moreover, data matching may be difficult because of different levels of detail of the data provided, requiring disambiguation/clarifiation of data types, which is a difficult process. Even for bioinformaticians, there is a great amount of ad hoc data processing which becomes quite a burden. Moreover, high-activity databases often update their data on a regular basis, often increasing the burden to continuously import the necessary information. Another burden lies in the need to develop individual query tools for each database, which may be limited in functionality and focus solely on the database at hand, still requiring researchers to integrate data from multiple sources manually.

In the midst of such activity, Linked Data has gained some attention recently in the life sciences as an effective way to provide and share data. As a part of the Semantic Web, data are linked so that a person or machine can explore the web of data. With Linked Data, when a user has some data, he/she can find other, related, data [1] rather easily. Resource Description Framework (RDF) is the standard means of implementing Linked Data. By using RDF, database providers can publish data contents that are accessible via URIs. In addition, each data contains links to other related data that are (preferably) provided in RDF. Thus, by crawling through the URIs that are linked to one another, a wide range of inter-related data can be retrieved using Semantic Web technologies. As an example, the Sindice portal provides a search engine to query RDF data across all domains. Using existing web standards, Sindice collects Semantic Web data, updated every five minutes, and allows users to search and query across this data [2].

In the process of generating RDF data, not only are data simply linked to one another, the links themselves are characterized by ontologies, thereby allowing the types of links to be distinguished. Although it may require a lot of effort for data providers to define an ontology, the merit lies in the higher level of interoperability with data analysis and visualization software. That is, related data are linked to one another via ontologies containing URIs, thus facilitating the multi-faceted retrieval of data, where the appropriate data can be quickly extracted and visualized. Such retrieval is usually performed using the SPARQL (SPARQL Protocol and RDF Query Language) query language, which is used against RDF data stores, or triplestores [3]. For the database provider, such interoperability will surely lead to an increase in the number of users.

This manuscript will describe the experiences and discussions shared among participants of BioHackathon 2011 who went through the development of RDF representations of their own data and developed specific RDF and SPARQL use cases within a period of five days. For bioinformaticians considering making data available and interoperable, this manuscript will provide advice regarding considerations to take when developing RDF representations of their data.

## Review

### Current landscape of Semantic Web in the life sciences

Linking Open Data (LOD) is a recent movement encouraging data providers to develop and to publish their data in a semantically connected manner. It is recommended that datasets are exposed and shared as Linked Data in RDF format, where URIs interlink resources on the Semantic Web. As shown in the LOD cloud diagram [4], life science data occupies one of the major domains of LOD. This situation is primarily brought by the Bio2RDF project [5] which translated major public bioinformatics databases into RDF and provided them as SPARQL endpoints. This pioneering work showed that distributed datasets in the life sciences can be effectively integrated through Semantic Web technology.

The semantics of RDF data is described by an ontology, which describes basic concepts in a domain and defines relations among them. It provides the basic building blocks comprising its structure: classes or concepts, properties, and restrictions on properties. As a result, an ontology provides a common vocabulary for researchers who need data integration, data sharing, semantic annotation, and extraction of information in the specific domain. To take advantage of Linked Data, one will eventually need to make use of ontologies. Several ontologies have already been carefully designed by experts in particular fields.

BioPortal is a useful web resource for developers to find a particular ontology in the life sciences. It provides an open repository and search engines for biological ontologies [6]. Moreover, the BioPortal Ontology Recommender system uses a set of keywords describing a domain of interest and suggests appropriate ontologies for representing the query [7]. The Open Biological and Biomedical Ontologies (OBO) Foundry provides biomedical ontologies, such as the well-known Gene Ontology (GO), with the goal of creating a suite of orthogonal interoperable reference ontologies in the biomedical domain [8].

The BioGateway project [9] attempts to query complex biological questions for obtaining scientific knowledge from RDF datasets in the semantic systems biology domain. They integrated SwissProt [10] protein annotations and taxonomic information with gene ontology annotations (GOA), ontologies provided by the open biological and biomedical ontologies (OBO) foundry and in-house developed ontologies such as cell cycle ontology (CCO). This system presented an example of how SPARQL queries can retrieve meaningful biological knowledge when the Semantic Web database contains rich information supported by fine-grained ontological annotations.

As the use of Semantic Web technologies increases, demand for SPARQL endpoints for major databases is raised. In response to these demands, UniProt has released their data in RDF and provides a publicly available SPARQL endpoint (http://beta.sparql.uniprot.org/). European Bioinforamtics Institute in the European Molecular Biology Laboratory (EMBL-EBI) recently started to provide RDF and SPARQL endpoints for several databases hosted at EBI (http://www.ebi.ac.uk/rdf/). BioMart [11] is one of the *de facto* standard databases integrating various resources in biology, and the system is widely used in many organizations [12]. A SPARQL query interface has been implemented since the version 0.8 release, enabling users to query the metadata of any BioMart system from Semantic web applications [13].

#### * LinkDB

LinkDB is a database that compiles relationships between database entries that have been serviced as the backbone of GenomeNet for nearly 20 years. As of August, 2011, a total of over 780,000,000 relationships between entries from over 160 life science databases have been registered. The data structure of LinkDB is triples, consisting of pairs of database entries and their relationships. Thus, it is very suitable for converting to RDF. The following three entry relationships are defined in LinkDB: equivalent (the same molecule but from different databases), original (hyperlinks to target database entry provided in the subject database entry), and reverse (opposite of original; subject database entry is referenced by target). These relationships could be used as predicates when generating RDF. During this BioHackathon, all of the LinkDB entries were converted to RDF. A manual describing how to use this data is available at http://www.genome.jp/linkdb/linkdb_rdf.html.

#### * PDBj

The Protein Data Bank Japan (PDBj), a member of the worldwide Protein Data Bank (wwPDB), is a database of atomic structures of proteins and other biological macromolecules. PDBj has recently started providing the contents of its entries in terms of RDF (http://rdf.wwpdb.org/). The RDF-formatted PDB entries are referred to as PDB/RDF in the following. The original PDB entries are provided in a format called macromolecular crystallographic information format (mmCIF), which is in turn defined by the PDB exchange (PDBx) dictionary [14, 15]. The PDBx dictionary defines categories and items for describing various aspects of macromolecular structures. The OWL ontology of the PDB/RDF is essentially a direct translation of the PDBx dictionary augumented with additional classes and properties to handle links between different data sources.

In the PDB/RDF service, each PDB entry can be accessed via a specific URL such as http://rdf.wwpdb.org/pdb/1GOF for the PDB entry 1GOF. This page contains mostly a list of links to the categories contained in the entry. By following these links, for example, http://rdf.wwpdb.org/pdb/1GOF/entityCategory, one finds a list of links to “entity” category elements. Each category element can be also accessed by a URL such as http://rdf.wwpdb.org/pdb/1GOF/entity/1 which contains the data describing the molecular entity whose the primary key is 1 (in this particular example, the entity is galactose oxidase. The PDBx dictionary also defines relations between related categories, and this is reflected in PDB/RDF as URL links between elements of different categories. In each PDB entry, there are also references to other resources such as UniProt, Enzyme Commission numbers, PubMed, DOI (document object identifier), etc. URL links to these resources are also included in PDB/RDF. Thus, the user agent can find information of various aspects of PDB entries by following the link structure of PDB/RDF.

#### * DDBJ

DNA Data Bank of Japan (DDBJ) is one of the members of International Nucleotide Sequence Database collaboration (INSDC). DDBJ has produced the database for two decades collaborating with GenBank/NCBI in the USA and EMBL-EBI in the UK. DDBJ has been opened to the public in a flat file format. However, the format is changeable and complex. For example, biological annotations are expressed in 957 pairs of feature and qualifier such as CDS feature and gene qualifier. The feature and qualifier names are revised by the meeting in INSDC every year.

Therefore DDBJ developed the initial version of RDF representation of DDBJ sequence records during BioHackahton 2011 which eliminates laborious parsing from users to extract biological annotations. Since then, it has been revised to incorporate an newly developed INSDC ontology which captures metadata of sequence entries and semantics among features and qualifiers. We also started to align the RDF data model with EBI so that the resulting data can be searched by the same SPARQL queries.

### Use cases

This section will describe two use cases involving the development of RDF data. Use case 1 describes the development of Linked Data pertaining to Alzheimer’s disease. Use case 2 describes the RDFization of glycobiology data.

### Use-case1: Alzheimer’s disease

In typical clinical microarray studies, differentially expressed genes are statistically identified between case and control. Among them, clinical researchers narrow down candidate genes responsible for pathogenesis by examining properties of their genes; e.g., functional annotations, metabolic and signaling pathways, and literature information. This narrowing-down process of candidate genes is an exploratory process. Clinical researchers examine candidate genes from as many angles (properties) as possible. If a novel property of genes become available and published on the web, clinical researchers incorporate it to investigate. Linked Data is suitable for this exploratory process. All the properties (data) linked to a gene can be retrieved by crawling Linked Data as RDF, and can be integrated and analyzed simply. In typical clinical microarray studies, clinical researchers spend time in this exploratory process. In this section, we propose a use case of application of Linked Data to microarray analysis in Alzheimer’s disease (AD) study as one of typical clinical microarray studies.

### Source data

Source data is (1) gene variation data (AlzGene), (2) gene expression data, (3) gene annotation data, (4) PubMed co-occurrence data. As for gene expression data, hippocampal gene expression of nine control and 22 Alzheimer’s disease subjects of varying severity (control, incipient, moderate, Severe) was used (NCBI GEO GSE1297). Annotation data provided by Affymetrix was used (HG-U133A.na31.annot.csv). As for PubMed co-occurrence data, a gene list co-occurred with “Alzheimer’s disease” in PubMed abstract was used.

### Data designing and transformation

#### (1) URI Scheme

HTTP URIs were adopted to identify biological entities. If original data providers provide their own URIs for their data, we used their original URIs. Otherwise, we used the Bio2RDF URIs because Bio2RDF is a pioneer in Linked Data in life science. As for URIs for Affymetrix probe sets, we chose Bio2RDF becase Affymetrix did not provide any URI for their probe sets and Bio2RDF provided URIs for Affymetrix probe sets.

#### (2) Unifying terms (predicates)

To realize federated searches, we used unified terms (predicates). If defined in Bio2RDF, we used Bio2RDF terms (predicates). If not, we defined our own terms (predicates). An example of one of our defined terms is “http://open-biomed.org/BH11Ujicha/ExprsFc_Severe_Control” which describes the fold change in expression values between severe and control samples. In RDF, it may not be appropriate to define such a context-specific predicate; it is better to use unifying terms (predicates) instead of our defined terms (predicates). See the section on Utilization of Ontologies in the Discussion for appropriate adoption of predicates.

#### (3) Ontology design

In this use case, we did not use any technical terms in neuroscience to represent data in RDF, however, if needed, we can utilize such ontologies. For example, the Neuroscience Information Framework (NIF) has developed a comprehensive ontology for describing neuroscience resources [16], and if needed, we can use NIF ontologies.

As for gene expression data, W3C Health Care and Life Sciences Interest Group (HCLSIG) examined the federation of microarray data and related data using the Linked Data technology. They examined and provided RDF representations of microarray data, and examined their use cases [17]. Also, EBI has started providing RDF representations of gene expression data in the Gene Expression Atlas on the RDF platform (http://www.ebi.ac.uk/rdf/services/atlas/) [18] since July 19, 2013. RDF representations of the NCBI GEO GSE1297 dataset are now provided in Gene Expression Atlas as the E-GEOD-1297 dataset. However, at the original time of this writing in 2011, the EBI RDF platform was not yet available so it could not be utilized.

**Figure 1:**
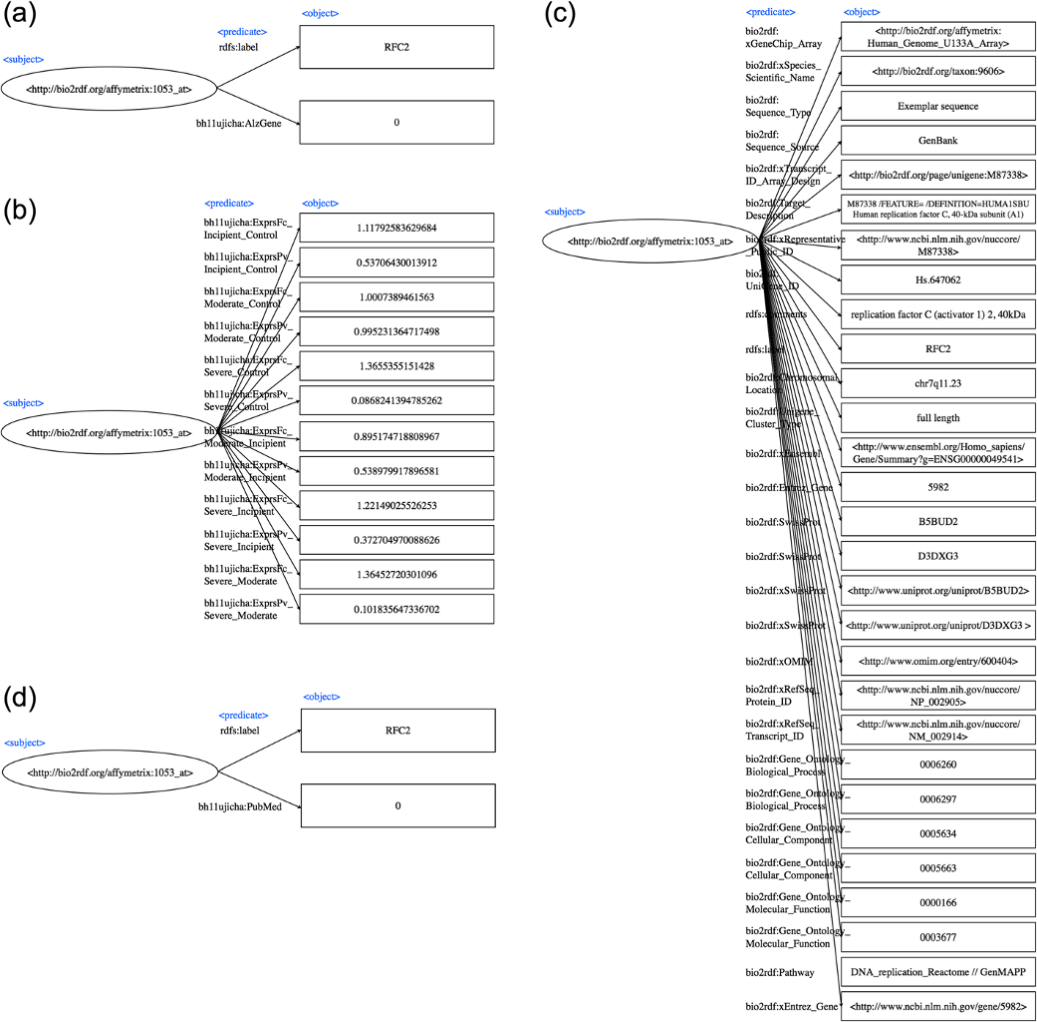
**(a) RDF diagram for gene variation data (AlzGene). (b)** RDF diagram for gene expression data (fold changes and P values). **(c)** RDF diagram for gene annotation data. **(d)** PubMed co-occurrence data.

According to the data design described above, source data was transformed to RDF in turtle format. A property and its corresponding value were transformed to a URI or literal as a subject and an object, respectively. We used Google Refine with RDF Extension for transformation. Details regarding the generated data is listed in Table 1. See RDF diagram of gene variation data (AlzGene), RDF diagram of gene expression data (fold changes and P values), RDF diagram of gene annotation data, RDF diagram of PubMed co-occurrence data for Figure 1(a), (b), (c), and (d), respectively.

**Table 1:** **Generated RDF of Alzheimer’s disease data**

Data type	URL of RDF (in turtle format) file	Number of triples
Gene variation data (AlzGene)	http://open-biomed.org/bh/11/ujicha/AlzGene.ttl	66856
Gene expression data (fold changes and P values)	http://open-biomed.org/bh/11/ujicha/GSE1297.ExprsFc.ttl	289686
Gene annotation data	http://open-biomed.org/bh/11/ujicha/HG-U133A.na31.annot.ttl	965966
PubMed co-occurrence data	http://open-biomed.org/bh/11/ujicha/Alzheimer_PubMed.ttl	66856
Total		1389364

### SPARQL query, facet view and crawling linked data

Suppose, for instance, an AD researcher can obtain his/her genes of interest, (1) showing differential expression between AD severe subjects and normal subjects, (2) functioning in the blood clotting cascade, (3) having the corresponding entry in OMIM [19], and (4) having the corresponding three-dimensional structure of coding proteins. After transforming source data to Linked Data in RDF, an AD researcher can obtain his/her genes of interest by both executing SPARQL query to SPARQL end point and crawling Linked Data.

### (1) SPARQL query and facet view

The following SPARQL query enables an AD researcher to obtain differentially expressed genes between AD severe subjects and normal subjects (Significance Analysis of Microarrays (SAM) P < 0.05; Fold Change > 1.5 or <0.67).



**Figure 2:**
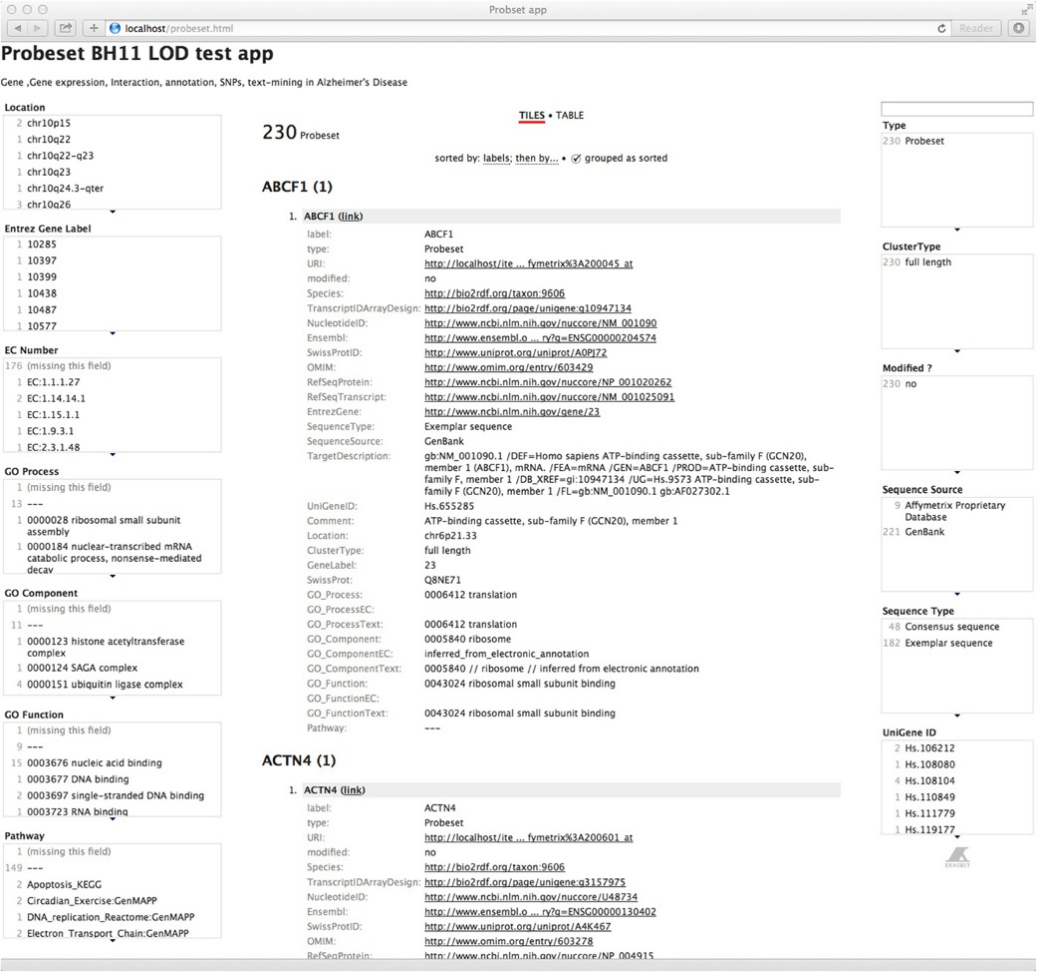
**Snapshot of the facet viewer app for the gene data of Alzheimer’s disease.**

The Virtuoso SPARQL endpoint returns 1824 differentially expressed genes (probe sets) and their relevant data (properties) in JSON format. As for these differentially expressed genes, we examined their properties using our facet viewer implemented by Exhibit library (Figure 2).

After examining candidate genes using the facet viewer, we decided to focus on differentially expressed genes on the blood clotting cascade. By executing the following SPARQL query, we obtained all the differentially expressed genes on the blood clotting cascade in the GenMAPP pathway database.



The Virtuoso SPARQL end point returns 4 differentially expressed genes (probe sets) and their relevant data (properties) as shown below in Table 2.

**Table 2:** **SPARQL results of differentially expressed genes in Alzheimer’s Disease data**

Probe set ID	Ratio	P value	Gene symbol	SwissProt URI
http://bio2rdf.org/affymetrix:204714_s_at	2.23	4.3 × 10^−3^	F5	http://www.uniprot.org/uniprot/B4DU26
http://bio2rdf.org/affymetrix:207218_at	2.01	3.7 × 10^−3^	F9	http://www.uniprot.org/uniprot/P00740
http://bio2rdf.org/affymetrix:209977_at	1.55	2.0 × 10^−2^	PLG	http://www.uniprot.org/uniprot/A6PVI2
http://bio2rdf.org/affymetrix:202112_at	0.60	4.2 × 10^−2^	VWF	http://www.uniprot.org/uniprot/A8K7V7

### (2) Traversing linked data

Among the genes that were narrowed down in Table 2, F9 (coagulation factor IX) showed the lowest P value, indicating that it is a plausible candidate responsible for pathogenesis. Next, we obtain data that can be possibly linked regarding Coagulation factor IX (http://www.uniprot.org/uniprot/P00740), which was obtained from the genes obtained in (1). For example, disease information regarding F9 (coagulation factor IX) is obtained from OMIM (http://omim.org/entry/300746). From this link traversal, it is possible to obtain the physiological and phenotypical information supporting the reason for selecting the plausible gene, which would normally be difficult to obtain from the gene’s functional annotation data alone.

Moreover, we can select plausible drug targets from the potential genes of (1). As a simple example, we extract those proteins whose structures have been solved together with a small molecule (based on a naive story whereby the inhibitor for this small molecule can be synthesized as a candidate drug). This can be done by the following steps. 1) we confirm whether the PDB ID exists in the RDF of the UniProt entry obtained by the previous SPARQL query. If this can be obtained, then 2) we confirm whether a small molecule is bound to this protein, from PDBj (whose RDF-ization is complete). Summary of traversing results from the four genes obtained in (1) are shown in Table 3.

**Table 3:** **PDB traversing results from the four genes obtained in (1)**

UniProt ID	Gene (protein) name	PDB entries with small compounds ^*^
B4DU26	cDNA FLJ50218, highly similar to Coagulation factor V	-
P00740	Coagulation factor IX (EC 3.4.21.22)	1RFN (PBZ), 3LC3 (IYX), 3LC5 (IZX)
A6PVI2	Plasminogen	-
A8K7V7	cDNA FLJ75522, highly similar to Homo sapiens von Willebrand factor (VWF), mRNA	-

^*^PDB entries are in 4-letter PDB ID, and small compounds are in 3-letter HETATM ID (in parentheses).

### Use-case 2: RINGS

#### (1) Background of glycobiology data

The RINGS web site [20] is a resource of data mining and analytical tools for understanding glycan function. Behind this resource is a database of glycan structures and related data such as lectins, glycolipids, glyco-genes, proteins (enzymes) and reactions. The data for this database were all manually integrated from various publicly available databases, such as KEGG GLYCAN [21], NCBI Gene [22], GlycomeDB [23], CAZy [24], Lectines [25], Animal Lectins DB [26], LipidBank [27], LIPID MAPS [28], and some from the literature. Then these data were organized into a MySQL database.

#### (2) Approaching the Semantic Web

In considering the transition to the Semantic Web, we considered the most efficient manner of making these data available and linkable, especially since many of the original databases are still detached from the Semantic Web. The main questions that arose were the following:

1. Should a triplestore be created, when we have spent so many resources to develop an RDB?
2. Do we need to develop an ontology first? What predicates should be used?
3. What URIs should be used?

For question (1), we learned from the PDBj group that although there is some software that can convert from RDB to RDF (i.e. D2RQ at http://d2rq.org/, ontop at http://ontop.inf.unibz.it/, R2RML at http://www.w3.org/TR/r2rml/), these were still in the developmental stage. It was suggested that RDF-formatted data be generated directly from the RDB. Thus, It was determined that as a first step, the data from the RDB would first be made available as REST web services in RDF format.

The second question of whether an ontology was necessary then went under consideration. For glycomics data, the GlycO ontology has been published online for some time [29]. To quote the description as stated in this OWL file:

The Glycomics Ontology GlycO focuses on the glycoproteomics domain to model the structure and functions of glycans and glycoconjugates, the enzymes involved in their biosynthesis and modification, and the metabolic pathways in which they participate. GlycO is intended to provide both a schema and a sufficiently large knowledge base, which will allow classification of concepts commonly encountered in the field of glycobiology in order to facilitate automated reasoning and information analysis in this domain.

This is a file with over 10000 lines and covers glycoconjugate structures at a very detailed level. However, the scope of the ontology was quite different from the RINGS data. For example, the glycan entries focused on describing the glycan structure in different formats and linking with the existing links found in KEGG GLYCAN, such as with KEGG REACTION, are not available in GlycO. The Lectin and Lipid data are also quite generalized, and it would require a glycochemist expert to properly annotate each of the entries with this ontology. Thus, we decided to put this ontology on hold and instead directly use the labels that were already being displayed via the existing web services as predicates.

Regarding question (3), for the object URIs, we learned about http://identifiers.org, which is a system providing resolvable persistent URIs used to identify data for the scientific community, with a focus on the Life Sciences domain, thus facilitating trustable URIs to be used for the Semantic Web and Linked Data. This site uses the resources as defined in MIRIAM [30]. Thus, as much as possible, we looked up the http://identifiers.org URI for each of our foreign links.

#### (3) RDFization of glycobiology data

**Figure 3:**
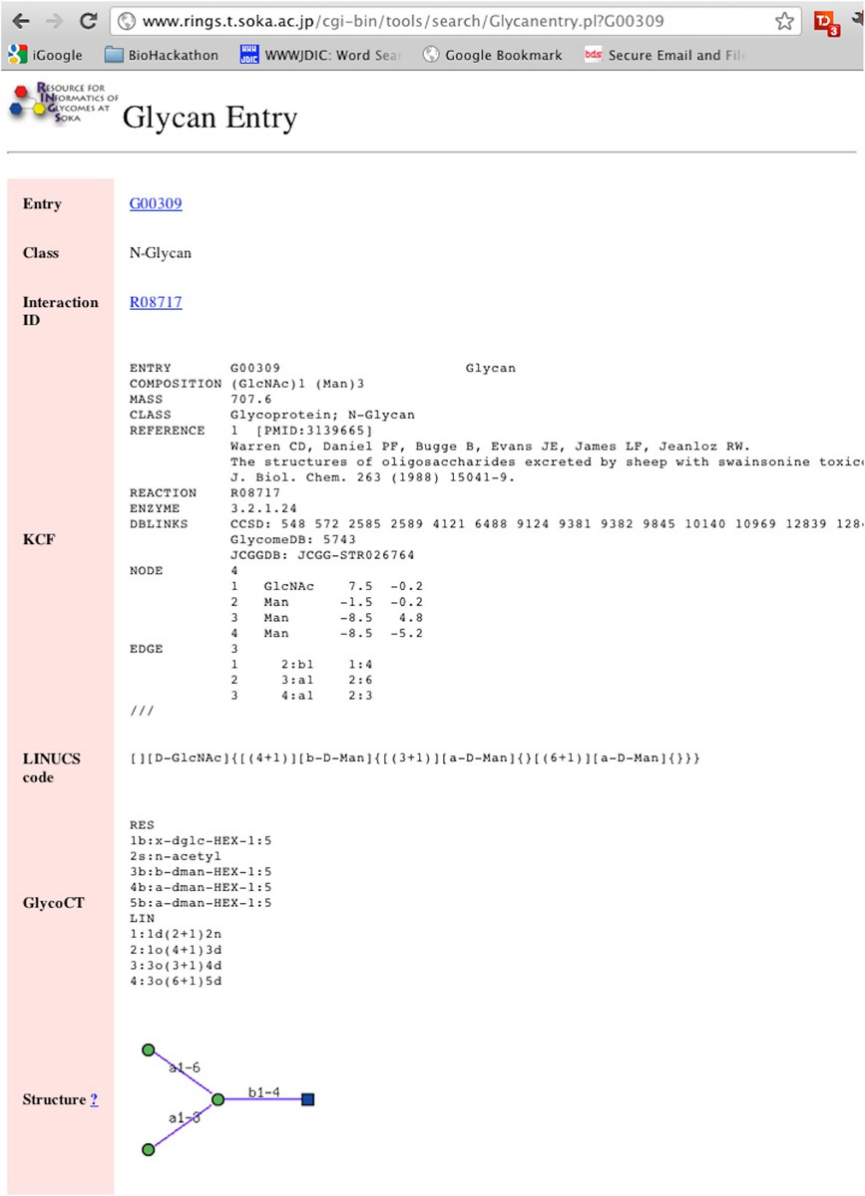
**An example of a glycan entry page in RINGS, describing the details of a particular glycan structure.** Originally sent in HTML format, such information can be RDF-ized by sending the corresponding data in RDF-XML. This can be done by transforming the data such that they are organized as triples (eg. turtle or n-triples format) which can be directly converted to RDF-XML.

The RINGS data is searchable from the main web page for a particular glycan, lectin or lipid entry, which is generated in a somewhat REST-like manner. For example, for glycan ID G00309, the following page (Figure 3) would be displayed as HTML in a web browser with the given URL containing the glycan ID.

So our first goal was to develop web services to return the same data in RDF format for particular glycan IDs, lectin IDs and lipid IDs. This was thus just a matter of rewriting our current code to generate RDF triples instead of HTML. An example of the resulting glycan entry G00309 in ntriples format is as follows:



Once this RDF data could be generated, then tools such as rapper [31] (http://librdf.org/raptor/rapper.html) can be used to generate RDF-XML from the triples, which could then be formatted appropriately for web browsers using xsl stylesheets. Although the triples themselves could be displayed in turtle format in the browser, for the glycobiologist, HTML was preferred.

For our next step, we will return to the GlycO ontology and make additions to this existing ontology with our RINGS data such that other database providers can use a consistent vocabulary in generating Linked Data in the future.

#### (4) Benefits of RDFization

From this use-case, we learned that it is possible to easily transform tables from RDB to RDF if we narrow the focus of the namespace. As described later, it will be important to incorporate an appropriate ontology in order to truly appreciate the utility of RDF data. The current RINGS namespace is limited to the local server, and the relationship of the data to other RDF data is unavailable. Moreover, the ontology is very simple. Extension of the ontology to describe substructures or patterns of glycan structures (known as “motifs” in the glycosciences) would make it more useful for performing structure searches of the database, for example. Once such RDF data is generated, the next step is to provide triplestores to house the data and allow federated queries to be made across various RDF databases. In other words, URIs referring to the same data entry (glycans in this case) can be easily linked to one another using RDF and queried together at once. This is in contrast to the current state of glycomics, where databases provide links to one another for each equivalent glycan, but the databases cannot be queried together. To make such queries available even for a single database, each database developer needed to write code to perform such queries on their databases. The reciprocal links also needed to be computed based on complicated tree-matching algorithms for glycans (in contrast to string comparisons often used for nucleotide or amino acid sequences). Thus by providing a centralized URI for glycan structures, duplicated efforts across different databases can be avoided. Moreover, by providing RDF data in triplestores, inference engines can be run on the data to glean new information from the linked data which could not be easily retrieved by browsing alone. The use of identifiers.org actually adds an extra step between the current data and the foreign link, which may cause some complications in federated queries. This is currently under discussion, but either an URI mapping or additional triples to the original URI may need to be incorporated in the RDF data.

## Discussion

The above use cases illuminate both the strength and weakness of the current status of Linked Open Data. Whereas with Web services, the usage of each service must first be checked before they can be used, with Linked Open Data, the most prominent strength is that users can simply access URIs and filter the retrieved data to obtain the relevant triples of interest. RDF data eliminates bothersome needs for struggling to develop specialized parsers and to find the correspondence between different databases because the links are already provided in the data themselves. In addition, individual data providers can define their ontologies for describing types and properties of data and publish their RDF independently of one another. This enables a truly distributed, global database or the Web of Data.

However, there are still several issues that should be addressed for the Semantic Web to be fully functional. In both use cases described in this work, RDF representations were newly created for each domain in order to accomplish their goals during the five-day BioHackathon. However, by putting more efforts into developing an ontology to cover the data at hand, mapping issues could be avoided later. Mapping fills in gaps between links that should refer to the same data, as described next.

### Missing links in linked data

A possible complication is that we need to know which triples are relevant to our purpose beforehand, that is, it is necessary to explicitly specify specific predicates and/or specific classes. Although it is assumed in the Semantic Web that the “meaning” of data is provided in the form of RDFS/OWL ontologies, those ontologies provide only formal structures and relationships between classes and properties and it is left for us to figure out, for example, which class actually corresponds to UniProt entries or Glycan entries. Currently, the greatest drawback is that there are still relatively few data providers that provide RDF data via dereferenceable URIs.

**Figure 4:**
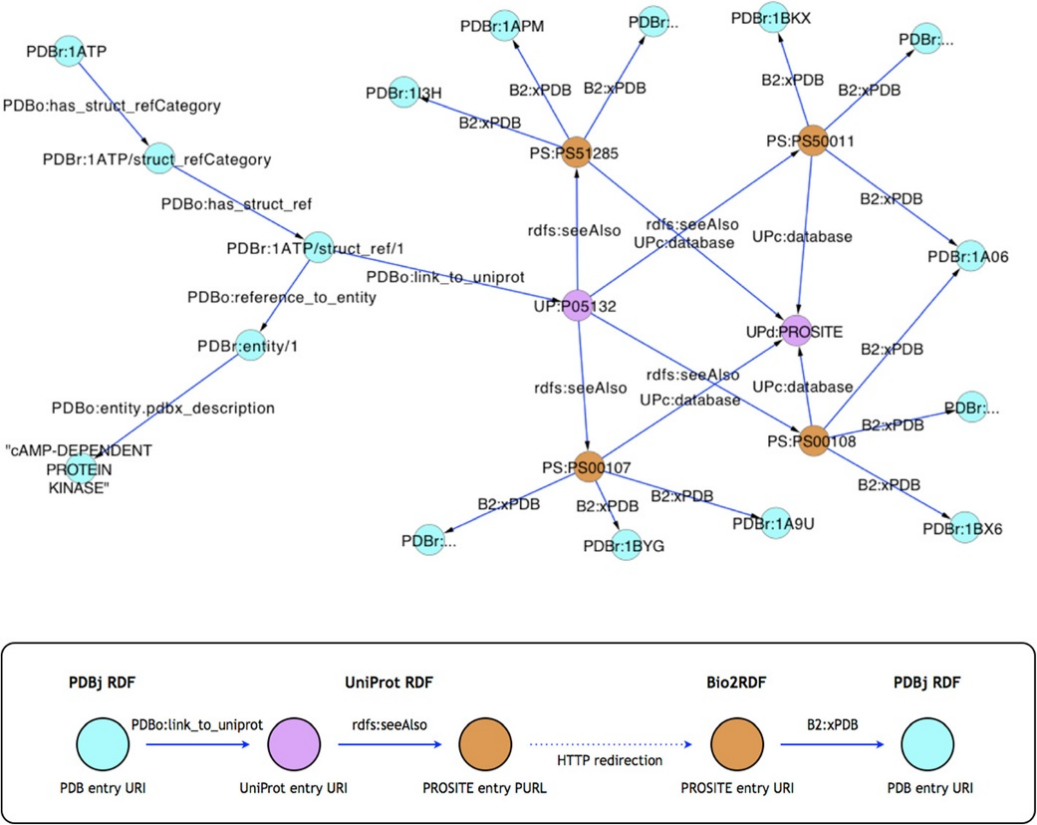
**Linked Data used in the use case.** Starting from a PDB entry PDBr:1ATP, links are followed through UniProt, PROSITE (Bio2RDF portal) to obtain other PDB entries sharing the same PROSITE motifs. Prefixes are defined as follow: PDBr: = http://pdbj.org/rdf/, UP: = http://purl.uniprot.org/uniprot/, PDBo: = http://rdf.wwpdb.org/schema/pdbx-v40.owl#, UPc: = http://purl.uniprot.org/core/, UPd: = http://purl.uniprot.org/database/PROSITE, PS: = http://purl.uniprot.org/prosite/ (this is transferred to http://bio2rdf.org/prosite:), B2: = http://bio2rdf.org/bio2rdf_resource:, rdfs: = http://www.w3.org/2000/01/rdf-schema#. The diagram was created using Cytoscape (Smoot, 2011).

For example, to find PDB entries which share the same PROSITE [32] sequence motifs in their protein sequences with a given PDB entry, we would find links from a PDBj [33] entry to UniProt [10] entries, then extract links to PROSITE entries, and finally follow the links back to other PDB entries. All these steps can be achieved simply by retrieving a series of URIs and filtering relevant triples, and iteration thereof. As shown in Figure 4, we started from a PDB entry of cAMP-dependent protein kinase <http://rdf.wwpdb.org/pdb/1ATP> (or PDBr:1ATP in short), which, after following some links, eventually reaches UniProt entry <http://purl.uniprot.org/uniprot/P05132> (or UP:P05132 in short). Next, by accessing UP:P05132, we find links to four PROSITE entries, PS00107, PS00108, PS50011, PS51285. Then, retrieving the PROSITE entries at the Bio2RDF service yields the final result, that is, the annotations of the PROSITE motifs and a list of PDB entries sharing those PROSITE motifs. However, there were several issues: (1) The PROSITE database does not provide RDF data at the time of this writing. (2) As a workaround, UniProt provides PURLs for PROSITE entries and redirects the PURLs to the corresponding entries in Bio2RDF (e.g., <http://purl.uniprot.org/prosite/PS00107> is redirected to <http://bio2rdf.org/prosite:PS00107>). Redirections from UniProt PURLs to Bio2RDF URIs are not provided as RDF data, so a SPARQL query will not resolve these links. The only option for a user is to access the PURLs and find the redirected URIs by following HTTP status codes such as 303 See Other and/or 302 Moved Temporarily. (3) Although Bio2RDF provides essential subsets of many databases, it is not targeted at providing full sets of all the databases. Nevertheless, as more data providers provide dereferenceable RDF data, the utility of Linked Data will increase tremendously.

### Utilization of ontologies

An ontology describes basic concepts in a domain and defines relations among them. It provides the basic blocks comprising its structure: classes or concepts, properties, and restrictions on properties. As a result, an ontology provides a common vocabulary for researchers who need data integration, data sharing, semantic annotation, and extraction of information in the specific domain. To take advantage of Linked Data, one will eventually need to make use of ontologies. Several ontologies have already been carefully designed by experts in particular fields, so whenever possible, researchers should try to use existing ontologies. Moreover, a variety of methodologies for building ontologies have been proposed [34, 35]. For those looking to use ontologies, the following three steps may be considered.

1. Ontology searching: As mentioned in the Review section, several ontology resources are currently available providing a number of ways to search for ontologies of interest, such as BioPortal and Sindice. Another tool called OntoFinder [36] displays a table with all matching ontologies in the columns and matching terms in the rows when searched. The base database is BioPortal, so it may take a while for results to display, but once the table is shown, users can freely filter down to their ontology of choice to find the most appropriate terms that match with the target terms.
2. Ontology merging: when existing ontologies are insufficient in terms of vocabulary and expressions, it is recommended to merge multiple related ontologies and/or remove unnecessary parts, creating a new ontology. Many editors have been developed for editing ontologies [37]. Protege is a major ontology editor, and WebProtege can support editing and full-fledged collaboration for domain experts [38, 39].
3. Ontology mapping: an ontology mapping is a correspondence between two ontologies, and mapping between different ontologies can enhance data linking on the Web. In Linked Data, use of owl:sameAs is ubiquitous in “inter-linking” data sets. However, the issue of how to represent relationships of identity on Linked Data is more complex than just applying owl:sameAs [40]. For terms that refer to types, it would be better to formalize them as classes, and make them equivalent using owl:equivalentClass. Ontologies such as SKOS (http://www.w3.org/TR/skos-reference/) also allow users to describe the relationships between ontologies and terms. For example, concepts such as “narrower” or “broader” can be used to describe the relationship between such terms.

Once an ontology has been developed, it should be published as an OWL file. Most ontology editors should support this format. Users may also consider submitting their ontology to BioPortal to allow it to be searched. This will allow other users to re-use the ontology to avoid recreating the same terms in a new ontology.

## Conclusions

With the rapid speed of growth of databases in number and volumes, there is a greater demand to integrate this data in order to interpret in-house data; that is, more agile data integration is sought. Thus, it would benefit users to provide a painless and flexible framework to facilitate the cycle of hypothesis construction and experimental validation. Linked Data is a step towards such a framework.

Linked Data with appropriate use of ontologies can link and integrate more datasets, thus increasing accessibility. The ultimate goal of being able to freely query Life Science data would then become a possibility. There are still many challenges such as to be able to make queries using a natural language (i.e. not only English, but also French, Italian, Japanese, etc.), and ensuring the accuracy of the data. The annual BioHackathons have allowed each project described in this review to get closer to this goal while keeping these challenges in mind. It is thus expected that in the near future, life science researchers will be able to retrieve their target information in an intelligent manner and with confidence.

We note that since the writing of this original manuscript, a similar manuscript describing emerging practices for mapping and linking life sciences data has been published, which may also be a good reference for beginners to this field [41].
